# Multilocus Family-Based Association Analysis of Seven Candidate Polymorphisms with Essential Hypertension in an African-Derived Semi-Isolated Brazilian Population

**DOI:** 10.1155/2012/859219

**Published:** 2012-09-26

**Authors:** L. Kimura, C. B. Angeli, M. T. B. M. Auricchio, G. R. Fernandes, A. C. Pereira, J. P. Vicente, T. V. Pereira, R. C. Mingroni-Netto

**Affiliations:** ^1^Centro de Estudos do Genoma Humano and Departamento de Genética e Biologia Evolutiva, Instituto de Biociências, Universidade de São Paulo, 11461 São Paulo, SP, Brazil; ^2^Laboratório de Genética Molecular, Departamento de Genética e Biologia Evolutiva, Instituto de Biociências, Universidade de São Paulo, SP, 11461 São Paulo, Brazil; ^3^Instituto do Coração (InCor), Faculdade de Medicina, Universidade de São Paulo, 11461 São Paulo, SP, Brazil; ^4^Departamento de Pediatria, Instituto da Criança, Hospital das Clínicas, Faculdade de Medicina, Universidade de São Paulo, 11461 São Paulo, SP, Brazil

## Abstract

*Background*. It has been widely suggested that analyses considering multilocus effects would be crucial to characterize the relationship between gene variability and essential hypertension (EH). *Objective*. To test for the presence of multilocus effects between/among seven polymorphisms (six genes) on blood pressure-related traits in African-derived semi-isolated Brazilian populations (*quilombos*). *Methods*. Analyses were carried out using a family-based design in a sample of 652 participants (97 families). Seven variants were investigated: *ACE* (rs1799752), *AGT* (rs669), *ADD*2 (rs3755351), *NOS*3 (rs1799983), *GNB3* (rs5441 and rs5443), and *GRK4* (rs1801058). Sensitivity analyses were further performed under a case-control design with unrelated participants only. *Results*. None of the investigated variants were associated individually with both systolic and diastolic BP levels (SBP and DBP, respectively) or EH (as a binary outcome). Multifactor dimensionality reduction-based techniques revealed a marginal association of the combined effect of both *GNB3* variants on DBP levels in a family-based design (*P* = 0.040), whereas a putative *NOS3-GRK4* interaction also in relation to DBP levels was observed in the case-control design only (*P* = 0.004). *Conclusion*. Our results provide limited support for the hypothesis of multilocus effects between/among the studied variants on blood pressure in *quilombos*. Further larger studies are needed to validate our findings.

## 1. Introduction

Common polymorphisms at multiple blood pressure-related loci from distinct biological pathways are likely to contribute to the genetic component of essential hypertension (EH) in humans [1–3]. Although previous evidence suggests that multilocus effects may account for a considerable proportion of the unexplained variance of complex traits, the role of multilocus effects in blood pressure traits still remains underexplored [1]. 

Approximately 4 million Africans were brought to Brazil as slaves over a period of four centuries. Before the abolition of slavery in Brazil (in 1888), many communities, named *quilombos* (presently known as *quilombo* remnants), were founded in isolated areas, in different Brazilian regions, by either runaway or abandoned African slaves [4–6]. Recent changes in lifestyle of current *quilombos*, such as a decrease in the intensity of agricultural activities and the nutrition transition, are associated with a larger frequency of common diseases such as obesity and essential hypertension [7, 8]

In this paper, we present the results of a carefully conducted family-based association study (652 participants from 97 informative families) in semi-isolated Brazilian populations of African ancestry, which includes individuals from remnants of *quilombos*. We hypothesized that multilocus effects between or among seven widely studied polymorphisms in six genes of major importance for blood pressure regulation might modulate the risk of essential hypertension in this peculiar population, characterized by historical inbreeding and high prevalence of cardiovascular risk factors [8].

## 2. Material and Methods

### 2.1. Population Investigated and Phenotyping

Participants were ascertained from African-derived Brazilian populations named *quilombos*, which encompass partially genetically isolated communities [9, 10]. These communities represent an interesting target group for the study of cardiovascular phenotypes, since recent changes in their lifestyle, such as a decrease in the intensity of agricultural activities and nutrition transition, are associated with a higher frequency of common diseases such as obesity and essential hypertension [7, 8]. Importantly, *quilombos *share a particularly homogeneous rural lifestyle and environment. Further information on these populations are found elsewhere [8–10].

Briefly, the sample consisted of individuals aged over 17 years with clinical and anthropometric data. Participants were sampled from 12 *quilombo *communities (Abobral, Galvão, São Pedro, Pedro Cubas, André Lopes, Nhunguara, Sapatu, Pilões, Ivaporunduva, Maria Rosa, Poça, and Reginaldo), located in the Vale do Ribeira region, São Paulo State (Southern Brazil). Figure S1 (of Supplementary Material available online at doi:40.1155/2012/859219.) presents the numbers of participants at each stage of the study.

Clinical evaluation occurred at multiple visits performed between 2003 and 2010. Due to the fact that *quilombos* are isolated populations, a detailed clinical evaluation was not feasible. However, participants with diabetes or suspected secondary forms of hypertension were excluded.

Blood pressure was measured after 15 min of rest in a sitting position. Two measurements were obtained by a physician from each participant at 5 min intervals. Systolic blood pressure (SBP) and diastolic blood pressure (DBP) values were means of the two physician-obtained measurements.

The primary phenotype was the risk of essential hypertension classified according to the World Health Organization (WHO) criteria (SBP ≥ 140 and/or DBP ≥ 90 and/or use of antihypertensive medication). Blood pressure was also evaluated as a continuous trait. In order to avoid shrinkage in estimated effects for continuous variables, blood pressure levels in participants taking antihypertensive drugs were adjusted by adding 15 mmHg and 10 mmHg to the SBP and DBP, respectively, as recommended previously [11].

Normotensive control participants were defined as those individuals with a SBP < 140 and DBP < 90 and no history of use of antihypertensive drugs. 

### 2.2. Genetic Variants Studied

We used the same rational described previously to choose the set of seven variants from six genes [8]. Briefly, the studied polymorphisms were chosen based on the fact that they represent examples of polymorphisms directly involved in blood pressure pathways, but that the cumulative evidence suggests either lack of effect or conflicting (i.e., heterogeneous) results across populations [12]. Our hypothesis was that these variants may not display individual effects, but might interact with each other in order to modulate the variance of blood pressure-related traits.

We hypothesized that seven variants from six major candidate genes directly involved in the (1) renin-angiotensin-aldosterone system: angiotensin-I-converting-enzyme (*ACE*, insertion/deletion in intron 16—rs1799752), and angiotensinogen (*AGT*, M235T—rs669); (2) sodium balance: *β*-adducin (*ADD2*, c.-154 + 20128C>A—rs3755351); (3) NO-dependent vasodilatation: nitric oxide synthase endothelial (*NOS3*, Glu298Asp—rs1799983); (4) intracellular signal transduction (*GNB3*, C825T—rs5443, and G-350A—rs5441); (5) dopaminergic system: G protein-coupled receptor kinase type 4 (*GRK4*, A486V—rs1801058) might be associated with blood pressure traits under a multilocus perspective. Further information on markers investigated is provided in Table S1 (online only material).

### 2.3. Genotype Determination

DNA was extracted from whole blood using standard procedures. Genotypes for the *Alu* insertion in the *ACE* were determined by polymerase chain reaction (PCR) and electrophoresis in agarose gels; all carriers of DD genotype (without *Alu* insertion) were genotyped twice. Genotypes for marker in the *GNB3* (G-350A) were determined by PCR followed by restriction fragment-length polymorphism analyses; all carriers of A allele were regenotyped by direct sequencing. The markers in *AGT*, *NOS3*,* GNB3 *(C825T), *ADD2, *and* GRK4* were genotyped following a minisequencing method using the *MegaBace SnuPe Genotyping Kit *(GE Healthcare, UK). Primers sequences are available as Supplementary Material (Table S1, online only material). Positive (previously tested samples with known genotype) and negative (buffer only) controls were included in each batch as a quality control measure. Genotyping was performed blinded to clinical status. 

### 2.4. Statistical Analysis

Data were expressed as means ± standard deviation (SD), median (interquartile range), or absolute number (percentage) when appropriate. The *t-*test for independent samples was used to investigate differences between groups for approximately normally distributed variables. When the distribution of the variables was skewed, the nonparametric Mann-Whitney *U *test was applied. Deviations from the Hardy-Weinberg equilibrium were tested by an exact approach [10]. 

#### 2.4.1. Strategy of Analysis

We performed two approaches of analysis: (i) a family-based design and (ii) an unrelated case-control design (Figure S1, online only material).

#### 2.4.2. Family-Based Analysis

Family-based association test (FBAT) analyses were performed with the FBAT program. The FBAT framework uses a variety of generalized linear models to perform tests similar to the transmission-disequilibrium test, allowing for the analysis of complex pedigrees [13]. We assumed the null hypothesis that there was no linkage and no association. For the risk of essential hypertension, FBAT analyses were run using (i) an offset value equal to zero (unadjusted), (ii) an offset values equal to the disease prevalence in the studied population (analysis with minimized variance), (iii) covariate-adjusted residuals (fully adjusted analysis). For continuous traits, we performed FBAT analyses considering (i) mean centered values (phenotypic mean approach), (ii) analysis with minimized variance (option –o, that is, an offset value that minimizes the variance of the FBAT statistic) and (iii) covariate adjusted-residuals (fully adjusted analysis). Main analyses assumed an additive model of action, whereas results for both dominant and recessive models were computed in sensitivity analyses.

Under a family-based design, we investigate all possible two-way multilocus effects using the recently proposed flexible family-based multifactor dimensionality reduction (FAM-MDR) approach [14]. The current framework of the FAM-MDR procedure focuses on potential multilocus associations for quantitative traits and can be schematically separated in two main stages. In the first stage, approximately normally distributed (family-based) traits are converted into independent, correlation family-free traits using a combination of mixed models and regression approaches. In the second stage, these new traits (residuals) are submitted to the model-based reduction technique (MD-MDR) yielding composite genotypes (i.e. for two loci, there are a total of nine possible composite genotypes) classified as *high risk*, *low risk* or *no evidence* based on the direction and magnitude of the association [15]. FAM-MDR analysis was carried out using a C++ program (available from the original authors). *P* values were computed by a resampling approach with 10,000 permutations. A full description of this technique is presented in detail elsewhere [14, 15].

#### 2.4.3. Analysis with Unrelated Participants

In sensitivity analyses, we also performed analysis considering 384 unrelated participants. For single-locus analysis, we used the MAX3 statistic which selects the largest test statistic from the dominant, recessive, and additive models [16]. To examine potential gene-gene interactions, we applied the generalized multifactor dimensionality reduction (GMDR) technique as described in detail elsewhere [17], whereas haplotypic analyses were computed according to the methodology described previously [18]. Data analysis was performed using the Stata 11.0 package (Stata, College Station, TX), R package (version 2.81, http://www.r-project.org/), THESIAS 3.1 (http://ecgene.net/genecanvas/), and GMDR (version 0.7, http://sourceforge.net/projects/gmdr/). Statistical significance was set at the 5% level (one-tailed for GMDR-based models and two-tailed for the remaining tests). 

## 3. Results 

### 3.1. Family-Based Study

From a total of 1521 potentially eligible inhabitants across 12 *quilombo* communities, we included 652 participants with complete data, representing approximately 43% of the total *quilombo* population. There were 97 informative families with an average number of subjects per family of 10.5 (ranging from 3 to 313). The largest family with 313 members represents a complex pedigree with a strong historical founder effect. This family has been partitioned accordingly in FBAT analysis.  Table 1 provides demographic and clinical for the 652 participants included in the family-based study. 

The prevalence of essential hypertension (EH) was 40.5% in women and 44.6% in men, a higher frequency when compared to admixed Brazilian populations [19–21] and African-derived populations [22]. No significant difference in the prevalence of hypertension was observed between genders (*P* = 0.169). 

#### 3.1.1. FBAT Analysis

In the single-locus FBAT analysis, no variant was significantly associated with any of the three examined blood pressure-related phenotypes, namely systolic and diastolic blood pressure (SBP and DBP, resp.) levels and EH (Table 2). FBAT-based haplotypic effects also did not show evidence for significant effects of both *GNB3* variants on blood pressure (data not shown).

#### 3.1.2. FAM-MDR Analyses

The family-based multifactor dimensionality reduction (FAM-MDR) method was applied to blood pressure as a continuous phenotype (i.e., both SBP and DBP in mmHg). As shown in Table 3, there was evidence for a marginally significant effect of both *GNB3 *variants (C825T and G-350A) on DBP levels (*P* = 0.04).

### 3.2. Case-Control Study 

We next performed a case-control study with unrelated subjects only, which yielded a total of 384 unrelated participants, whose clinical characteristics are shown in Table 4. 

#### 3.2.1. Single-Locus Associations

Table 5 shows the distribution of genotypes and allele frequencies for the seven studied polymorphisms according to hypertension status. There is no evidence of association of any studied variant with hypertension. Similar results were observed for blood pressure as a continuous variable (e.g., SBP and DBP, data not shown).

#### 3.2.2. GNB3 Haplotypic Effects

Haplotypic analyses of both G-350A and C825T *GNB3* variants based on unrelated participants (Table 6) indicated that the haplotype T825/G-350 might be associated with the odds of essential hypertension (OR = 0.586, 95%  CI = 0.358–0.958), although the alternative hypothesis of a better fit for the alternative model (e.g., a model with significant haplotypic effects) failed to be accepted (*P* = 0.152).

#### 3.2.3. Multilocus Associations

GMDR-based models were constructed to exhaustively identify all possible two- to four-locus models that potentially have an influence on the risk of hypertension and/or modulate SBP and DBP levels in *quilombos* (Table 7). These analyses revealed a single two-locus effect between the *NOS3* and *GRK4* variants on DBP levels that was statistically significant (*P = *0.0044) and had a high cross-validation consistency (90%).

## 4. Discussion

### 4.1. Findings

It has been shown that analyses focusing on biologically plausible candidate genes might be a strategy to circumvent the problem of limited coverage of current genome-wide association (GWA) platforms. This strategy holds the promise of increasing the density of markers in regions coding for known blood pressure-related effectors that are likely to be missed by GWA studies in hypertension [23].

 Here, we present a comprehensive investigation about the potential role of seven variants (from six candidate genes directly involved in the blood pressure regulation) in the susceptibility of essential hypertension in a semi-isolated, African-derived population (*quilombos*). As described previously, *quilombos* may be an important target population for genetic studies of complex diseases, since this population displays peculiar genetic and environmental characteristics [8]. Specifically, *quilombos *are known not only to have a large contribution of African genes, but also strong founder effects, a substantial degree of inbreeding, and high prevalence of essential hypertension as well as overweight [7–10, 24].

Using different methodological approaches (single-locus, haplotypic, and multilocus effects) and study designs (family-based and unrelated case-control designs), our investigation did not suggest an important contribution of the studied markers in the risk of essential hypertension in *quilombos*. However, our data highlight the potential of two multilocus effects: (i) the effect of both *GNB3* variants (C825T and G-350A polymorphisms) on DBP levels in a family-based design and (ii) the significant *NOS3-GRK4 *interaction also in relation to DBP levels in the unrelated case-control design. 

Meta-analyses addressing the role of the C825T polymorphism at the *GNB3 *locus in hypertension provide evidence for heterogeneity in the genetic effects across populations [25] (i.e., effects found in different populations vary in a larger extent from what would be expected by chance alone) [12]. However, the overall evidence suggests a weak, but significant association of 825T allele with an augmented odds of essential hypertension in both European [25] and Chinese populations [26]. Although no systematic evidence exists for the G-350A variant, hints from previous investigations corroborate to the hypothesis of synergistic effects between and among *GNB3 *variants on blood pressure levels [27]. Thus, our observations of a putative C825T/G-350A influence on blood pressure levels may serve as a hypothesis-generating finding for further in-depth investigations.

Recently, gene-centric approaches have revealed *NOS3* markers as significant factors influencing blood pressure levels [28]. These observations corroborate previous investigations, which indicated the potential of role of genetic variants in the nitric oxide pathway on the susceptibility of essential hypertension [2, 29]. Interestingly, among all two- to four-locus interactions, the *NOS3*-*GRK4 *model was the only combination found to achieve nominal significance with an adequate cross-validation consistency. Recently, despite the use of different markers, the *NOS3*-*GRK4* synergistic effect on blood pressure was also observed in an African American sample [30], suggesting that our result may be a genuine association signal rather than a chance finding. The G-protein-coupled receptor kinase 4 (GRK4) is involved in the regulation of sodium balance [31], and genetic variations at the *GRK4 *are associated with both impaired natriuresis and salt-sensitive hypertension [31, 32]. Thus, a potential biological mechanism underlying the statistical finding from the *NOS3*-*GRK4 *model might be the cumulative effect from both pathways. Indeed, a diminished natriuresis along with an impaired endothelium-dependent (i.e., NOS3-dependent) vasodilatation might lead to increased blood pressure levels. Nonetheless, the plausibility of the observed multilocus effects along with its generalizability across different populations requires further and detailed investigations.

### 4.2. Limitations

Major limitations of our study include: first, the small number of participants investigated, that is, our samples are underpowered to detect typical main effects of genetic variants associated with common traits and second, lack of information on serum lipids as well as accurate information on smoking habits. In fact, lipids were not investigated due to technical restrictions. Furthermore, we observed that there was inaccurate reporting of smoking status by participants mainly due to heterogeneity in the definition of smoking. For example, a high proportion of participants reported to smoke homemade cigarettes, but failed to consider this as a “smoking habit”. As a result, both lipids and smoking status were not included in adjusted analyses.

Furthermore, our results should be carefully interpreted because correction for multiple testing was not performed in our analyses. As this is a hypothesis testing study, we considered that correction for multiple testing would be overly conservative and might lead to a substantial loss of statistical power.

Finally, it is worth of mentioning that the present study does not invalidate the hypothesis of gene-gene interactions/multilocus effects between or among the studied variants nor single-loci effects on blood pressure. Our null associations might plausibly be associated with low statistical power and the reduced panel of markers investigated. 

## 5. Conclusions

In conclusion, our results do not support the hypothesis of significant association between the seven investigated variants and the risk of essential hypertension in *quilombos* but highlight potential multilocus association signals that should be investigated in further studies with larger samples.

## Supplementary Material

The supplementary material includes one figure (Figure S1) and one Table (Table S1). Figure S1 shows all the steps in the selection of subjects for the different types of analyses, both family-based studies and case-control studies. Table S1 provides complete information about the molecular markers selected for association studies with phenotypes related to hypertension, including nucleotide sequences of primer pairs and genomic identification of the polymorphisms.

## Figures and Tables

**Table 1:** Descriptive statistics of 652 individuals from 12 *quilombo* populations regarding to studied phenotypes.

*N* = 652	
Age (years)	43.5 (17–91)
Male (%)	45.6%
SBP (mmHg)	131.8 ± 25.6
DBP (mmHg)	82 ± 13.7
Hypertension*	41.6%
BMI (Kg/m^2^)	24.7 ± 4.48
BMI > 25 Kg/m^2^	41.9%

*Hypertension was defined as a SBP ≥ 140 mmHg and/or a DBP ≥ 90 mmHg or use of antihypertensive medications.

**Table 2:** FBAT analysis for blood pressure-related traits in *quilombos*.

Gene	Variant	rs	*F*		Phenotypes		
					EH	SBP	DBP
*ACE*	I/D	rs1799752	0.50	NIF	97	93	95
				*P* value	0.774	0.155	0.123
*NOS3*	Glu298Asp	rs1799983	0.16	NIF	63	63	61
				*P* value	0.101	0.050	0.079
*GNB3*	C825T	rs5443	0.58	NIF	86	84	82
				*P* value	0.793	0.417	0.342
*GNB3*	G-350A	rs5441	0.68	NIF	79	76	76
				*P* value	0.063	0.672	0.565
*AGT*	M235T	rs699	0.27	NIF	96	92	94
				*P* value	0.608	0.293	0.579
*ADD2*	c.-154+20128C>A	rs3755351	0.34	NIF	97	93	95
				*P* value	0.196	0.209	0.137
*GRK4*	A486V	rs1801058	0.24	NIF	70	69	68
				*P* value	0.096	0.229	0.230

NIF: number of informative families. *F*: allele frequency for the risk allele. EH: essential hypertension. SBP: systolic blood pressure (mmHG). DBP: diastolic blood pressure (mmHg). All analyses were adjusted for covariates (age, gender, and BMI when appropriate). Similar results were obtained for other FBAT approaches (e.g. prevalence adjusted, mean centered, or minimal variance approaches) (data now shown). All results refer to analyses performed under assumption of an additive model of action. Qualitatively analogous results were observed for other genetic models (e.g., dominant and recessive) (data not shown).

**Table 3:** Best two-locus models from FAM-MDR analyses.

Phenotype	Model	*F* test	*P* value
SBP	*GNB3*(C825T), *ADD2 *	8.203	0.533
	*GNB3*(C825T), *ACE *	6.318	0.819
	*GNB3*(G-350A), *GRK4 *	6.043	0.837
	*GNB3*(G-350A), *ACE *	4.172	0.981
	*ACE*, *ADD2 *	3.864	0.984
	*NOS3*, *ADD2 *	3.146	0.997

DBP	*GNB3*(C825T), *GNB3*(G-350A)	14.064	0.040
	*GNB3*(C825T), *ACE *	8.500	0.460
	*GNB3*(G-350A), *ADD2 *	8.014	0.512
	*GNB3*(G-350A), *ACE *	7.970	0.512
	*NOS3*, *ADD2 *	6.763	0.681
	*GNB3*(C825T), *ADD2 *	6.364	0.732

SBP: systolic blood pressure. DBP: diastolic blood pressure.

**Table 4:** Clinical characteristics of the unrelated *quilombo* sample.

	Normotensives (*n* = 206)	Hypertensives (*n* = 178)	*P* value
Gender			
Men	98 (47.57)	79 (44.38)	0.540
Women	108 (52.43)	99 (55.62)	
Age (years)	32 (24.5–44.7)	55.7 (42.4–67.4)	<0.001
Adjusted SBP (mmHg)^∗^	115.54 ± 12.29	155.85 ± 21.39	<0.001
Adjusted DBP (mmHg)^∗^	74.79 ± 7.22	94.84 ± 13.60	<0.001
BMI (Kg/m^2^)	23.95 ± 3.89	25.79 ± 4.40	<0.001

^∗^Adjusted for the antihypertensive use according the proposition by Tobin et al. [11].

**Table 5:** Genotype frequencies for the seven studied polymorphisms in normotensive and hypertensive *quilombo *subjects.

Gene/Status	Genotype, *n* (%)			*P* HWE	*F*	*P* MAX3
*ACE *(I/D)	I/I	I/D	D/D			
Hypertensive	39 (21.91)	92 (51.69)	47 (26.40)	0.474	0.518	0.227
Normotensive	54 (26.21)	92 (44.66)	60 (29.13)			
*NOS3 *(Glu298Asp)	Glu/Glu	Glu/Asp	Asp/Asp			
Hypertensive	127 (71.35)	47 (26.40)	4 (2.25)	0.842	0.150	0.922
Normotensive	151 (73.30)	50 (24.27)	5 (2.43)			
*GNB3 *(C825T)	C/C	C/T	T/T			
Hypertensive	42 (23.60)	74 (41.57)	62 (34.83)	0.999	0.592	0.054
Normotensive	22 (10.68)	111 (53.88)	73 (35.44)			
*GNB3* (G-350A)	G/G	A/G	A/A			
Hypertensive	93 (52.25)	60 (33.71)	25 (14.04)	0.028	0.297	0.595
Normotensive	106 (51.46)	82 (39.81)	18 (8.74)			
*AGT* (M235T)	Thr/Thr	Thr/Met	Met/Met			
Hypertensive	91 (51.12)	73 (41.01)	14 (7.87)	0.425	0.259	0.482
Normotensive	123 (59.71)	68 (33.01)	15 (7.28)			
*ADD2 *(A/C)	A/A	A/C	C/C			
Hypertensive	27 (15.17)	94 (45.63)	83 (46.63)	0.256	0.661	0.565
Normotensive	22 (10.68)	68 (38.20)	90 (43.69)			
*GRK4 *(A486V)	Ala/Ala	Ala/Val	Val/Val			
Hypertensive	98 (55.06)	72 (40.45)	8 (4.49)	0.893	0.253	0.586
Normotensive	117 (56.80)	72 (34.95)	17 (8.25)			

HWE: Hardy-Weinberg equilibrium; *F*: frequency of the risk allele; MAX3: max-statistic; MAX3-based results are adjusted for age, gender, and BMI.

**Table 6:** Analysis of haplotypic effects (G-350A/C825T) on blood pressure-related phenotypes.

Trait	*N*	Haplotypes		Haplotypic effects		LD measures		LR test	
		G(-350A)	C825T	OR (95% CI)	*P* value	*D* ^'^	*R* ^2^	*χ* ^2^ (df)	*P* value
Hypertension	384	G	C	reference	—	0.40	0.10	5.29 (3)	0.152
		G	T	0.586 (0.358–0.958)	0.033				
		A	C	0.591 (0.336–1.040)	0.068				
		A	T	0.865 (0.451– 1.659)	0.661				

				Δ (95% CI)	*P* value				

SBP (mmHg)	384	G	C	reference	—	0.40	0.10	7.04 (3)	0.071
		G	T	−2.208 (−6.734–2.318)	0.339				
		A	C	−0.482 (−5.95–4.986)	0.862				
		A	T	−1.361 (−7.39–4.67)	0.658				

				Δ (95% CI)	*P* value				

DBP (mmHg)	384	G	C	reference	—	0.40	0.10	2.82 (3)	0.420
		G	T	−1.247 (−3.878–1.383)	0.352				
		A	C	−2.328 (−5.447–0.792)	0.143				
		A	T	−1.996 (−5.596–1.605)	0.277				

*N*: sample size. OR: odds ratio. EH: essential hypertension. LD: linkage disequilibrium. LR: likelihood ratio. df: degrees of freedom. Δ: expected linear increment in the trait per additional copy of the haplotype (in mmHg). All results refer to the additive model of analysis and are adjusted for age, gender, and BMI. Analyses were also performed under both dominant and recessive models of action, yielding qualitatively analogous results (data not shown). The overall proportion of haplotypes G-350A/C825T in the studied population was 0.197, 0.499, 0.196, and 0.108 for haplotypes GC, GT, AC, and AT, respectively. For the hypertension status, haplotypic frequencies for hypertensive/normotensive participants were as follows: GC: 0.248/0.186, GT: 0.443/0.523, AC: 0.195/0.190 and AT: 0.114/0.10.

**Table 7:** Best predictive models from GMDR analyses.

Model	Test accuracy	CVC	*P* value
	Hypertension		
*GNB3(G-350A), GRK4*	0.4884	4	0.5324
*NOS3, GNB3(C825t), ADD2*	0.5569	4	0.0792
*GNB3(C825t), GNB3(G-350A), AGT, GRK4*	0.5850	3	0.0222

	Systolic blood pressure		
*GNB3(G-350A), GRK4*	0.5220	5	0.2644
*NOS3, GNB3 (G-350A), AGT*	0.5752	5	0.0444
*ACE, GNB3(G-350A), ADD2, GRK4*	0.5282	4	0.2440

	Diastolic blood pressure		
*NOS3, GRK4*	0.6031	9	0.0044
*ACE, ADD2, GRK4*	0.5677	3	0.0724
*ACE, NOS3, GNB3(C825t), GRK4*	0.6347	6	0.0014

CVC: cross-validation consistency.

## References

[1] Williams SM, Ritchie MD, Phillips JA (2004). Multilocus analysis of hypertension: a hierarchical approach. *Human Heredity*.

[2] Pereira TV, Rudnicki M, Cheung BMY (2007). Three endothelial nitric oxide (NOS3) gene polymorphisms in hypertensive and normotensive individuals: meta-analysis of 53 studies reveals evidence of publication bias. *Journal of Hypertension*.

[3] Pereira TV, Nunes ACF, Rudnicki M, Yamada Y, Pereira AC, Krieger JE (2008). Meta-analysis of the association of 4 angiotensinogen polymorphisms with essential hypertension: a role beyond M235T?. *Hypertension*.

[4] Ribeiro GGBL, Abe-Sandes K, Barcelos RDSS, Klautau-Guimarães MDN, Junior WADS, Oliveira SFD (2011). Who were the male founders of rural Brazilian Afro-derived communities? A proposal based on three populations. *Annals of Human Biology*.

[5] Lopes Maciel LG, MRibeiro-Rodrigues E, Carneiro Dos Santos NP, Ribeiro-dos-Santos Â, Guerreiro JF, Santos S (2011). Afro-derived Amazonian populations: inferring continental ancestry and population substructure. *Human Biology*.

[6] Da Silva WA, Bortolini MC, Meyer D (1999). Genetic diversity of two African and sixteen South American populations determined on the basis of six hypervariable loci. *American Journal of Physical Anthropology*.

[7] Ferreira HS, Lamenha ML, Xavier Junior AF, Cavalcante JC, Santos AM (2011). Nutrition and health in children from former slave communities (*quilombos*) in the state of Alagoas, Brazil. *Revista de Saúde Pública*.

[8] Angeli CB, Kimura L, Auricchio MT (2011). Multilocus analyses of seven candidate genes suggest interacting pathways for obesity-related traits in Brazilian Populations. *Obesity*.

[9] Mingroni-Netto RC, Angeli CB, Auricchio MTBM (2002). Distribution of CGG repeats and FRAXAC1/DXS548 alleles in South American populations. *American Journal of Medical Genetics*.

[10] Auricchio MTBDM, Vicente JP, Meyer D, Mingroni-Netto RC (2007). Frequency and origins of hemoglobin S mutation in African-derived Brazilian populations. *Human Biology*.

[11] Tobin MD, Sheehan NA, Scurrah KJ, Burton PR (2005). Adjusting for treatment effects in studies of quantitative traits: antihypertensive therapy and systolic blood pressure. *Statistics in Medicine*.

[12] Pereira TV, Patsopoulos NA, Salanti G, Ioannidis JPA (2009). Discovery properties of genome-wide association signals from cumulatively combined data sets. *American Journal of Epidemiology*.

[13] Laird NM, Horvath S, Xu X (2000). Implementing a unified approach to family-based tests of association. *Genetic Epidemiology*.

[14] Cattaert T, Urrea V, Naj AC (2010). FAM-MDR: a flexible family-based multifactor dimensionality reduction technique to detect epistasis using related individuals. *PLoS ONE*.

[15] Calle ML, Urrea V, Vellalta G, Malats N, Steen KV (2008). Improving strategies for detecting genetic patterns of disease susceptibility in association studies. *Statistics in Medicine*.

[16] González JR, Carrasco JL, Dudbridge F, Armengol L, Estivill X, Moreno V (2008). Maximizing association statistics over genetic models. *Genetic Epidemiology*.

[17] Lou XY, Chen GB, Yan L (2007). A generalized combinatorial approach for detecting gene-by-gene and gene-by-environment interactions with application to nicotine dependence. *American Journal of Human Genetics*.

[18] Tregouet DA, Garelle V (2007). A new JAVA interface implementation of THESIAS: testing haplotype effects in association studies. *Bioinformatics*.

[19] Ferreira SRG, de Moura EC, Malta DC, Sarno F (2009). Frequency of arterial hypertension and associated factors: Brazil, 2006. *Revista de Saúde Pública*.

[20] Piccini RX, Facchini LA, Tomasi E (2012). Promotion, prevention and arterial hypertension care in Brazil. *Revista de Saúde Pública*.

[21] de Oliveira CM, Pereira AC, de Andrade M, Soler JM, Krieger JE (2008). Heritability of cardiovascular risk factors in a Brazilian population: Baependi Heart Study. *BMC Medical Genetics*.

[22] Hall JL, Duprez DA, Barac A, Rich SS (2012). A review of genetics, arterial stiffness, and blood pressure in african americans. *Journal of Cardiovascular Translational Research*.

[23] Johnson AD, Newton-Cheh C, Chasman DI (2011). Association of hypertension drug target genes with blood pressure and hypertension in 86 588 individuals. *Hypertension*.

[24] Cotrim NH, Auricchio MTBM, Vicente JP, Otto PA, Mingroni-Netto RC (2004). Polymorphic Alu insertions in six Brazilian African-derived populations. *American Journal of Human Biology*.

[25] Bagos PG, Elefsinioti AL, Nikolopoulos GK, Hamodrakas SJ (2007). The GNB3 C825T polymorphism and essential hypertension: a meta-analysis of 34 studies including 14 094 cases and 17 760 controls. *Journal of Hypertension*.

[26] Niu W, Qi Y (2011). Association of *α*-adducin and G-protein *β*3 genetic polymorphisms with hypertension: a meta-analysis of Chinese populations. *PLoS ONE*.

[27] Li B, Ge D, Wang Y (2005). G protein *β*3 subunit gene variants and essential hypertension in the northern Chinese Han population. *Annals of Human Genetics*.

[28] Johnson T, Gaunt TR, Newhouse SJ (2011). Blood pressure loci identified with a gene-centric array. *American Journal of Human Genetics*.

[29] Kitsios GD, Zintzaras E (2010). An NOS3 haplotype is protective against hypertension in a Caucasian Population. *International Journal of Hypertension*.

[30] Martinez Cantarin MP, Ertel A, Deloach S (2010). Variants in genes involved in functional pathways associated with hypertension in African Americans. *Clinical and Translational Science*.

[31] Harris RC (2012). Abnormalities in renal dopamine signaling and hypertension: the role of GRK4. *Current Opinion in Nephrology and Hypertension*.

[32] Yatabe J, Yatabe MS, Yoneda M (2012). Hypertension-related gene polymorphisms of G-protein-coupled receptor kinase 4 are associated with NT-proBNP concentration in normotensive healthy adults. *International Journal of Hypertension*.

