# Supplementary figures and images for: Multilocus Family-Based Association Analysis of Seven Candidate Polymorphisms with Essential Hypertension in an African-Derived Semi-Isolated Brazilian Population
Source: Int J Hypertens. 2012 Sep 26;2012:859219. doi: 10.1155/2012/859219 (PMC3463917; doi:10.1155/2012/859219)

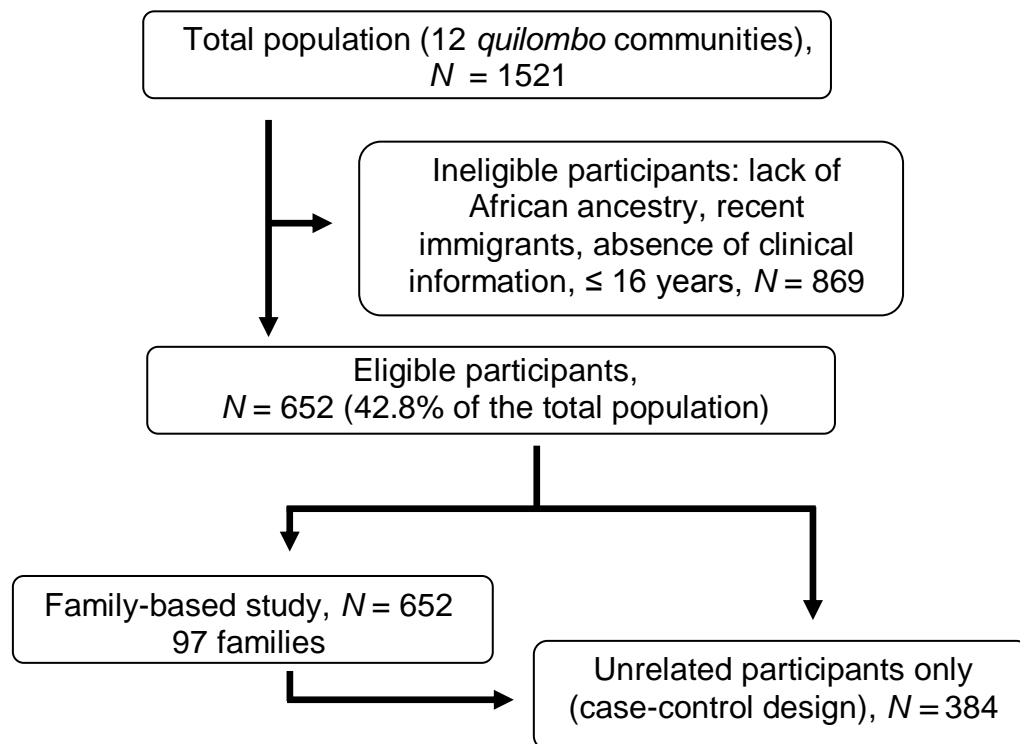

Figure S1. Flow diagram showing the key steps in the selection of participants.

Supplement: Supplementary file 1 — The supplementary material includes one figure (Figure S1) and one Table (Table S1). Figure S1 shows all the steps in the selection of subjects for the different types of analyses, both family-based studies and case-control studies. Table S1 provides complete information about the molecular markers selected for association studies with phenotypes related to hypertension, including nucleotide sequences of primer pairs and genomic identification of the polymorphisms. [file 859219.f1.pdf]
