# Supplementary material for: Multilocus Family-Based Association Analysis of Seven Candidate Polymorphisms with Essential Hypertension in an African-Derived Semi-Isolated Brazilian Population
Source: Int J Hypertens. 2012 Sep 26;2012:859219. doi: 10.1155/2012/859219 (PMC3463917; doi:10.1155/2012/859219)
Supplement: Supplementary file 2 [file 859219.f2.pdf]

Table S1: Information on the seven markers investigated and primers sequences.

**Gene**	**SNP**	**dbSNP**	**Chr**	**Alleles**	***Primers***	***** Snupe Primer*** **/*Restriction site**
ACE	I/D	rs1799752	17q23	I/D	F - CTGGAGACCACTCCCATCCTTTCT<br>R - GATGTGGCCATCACATTCGTCAGAT	
ADD2	c.-154+20128C>A	rs3755351	2p13	A/C	F – AGAGCAAATGCCTTTGTACCAT<br>R – CCATCATGCTATATGCCTTCCT	**GGCTCCCAAGTCACTAGTCC
AGT	M235T	rs669	1q42-q43	C/T	F – ACCTGAAGCAGCCGTTTGT<br>R - CAGGGTGCTGTCCACACTGGACCCC	**GAAGACTGGCTGCTCCCTGA
GNB3	C825T	rs5443	12p13	C/T	F - CTTGCCCTGGAGCTGTCA<br>R - CTTCCAGCTGAGGAAGCAG	**ATCATCTGCGGCATCACGTC
GNB3	G-350A	rs5441	12p13	A/G	F – CTGGACAGATGGGGAACACT<br>R – CTGACAAATCACAGGCTCCA	*TaqI
NOS3	Glu298Asp	rs1799983	7q36	G/T	F – GAAGGCAGGAGACAGTGGAT<br>R – CAGCAGCATGTTGGACACTG	**CTGCTGCAGGCCCCAGATGA
GRK4	A486V	rs1801058	4p16	C/T	F – ACTAAAAGCTGGCGAACCAC<br>R - TGGAAGGGCAGTACCTCATT	**GGATATCGAGCAGTTCTCGG
